# Artesunate, imatinib, and infliximab in COVID‐19: A rapid review and meta‐analysis of current evidence

**DOI:** 10.1002/iid3.628

**Published:** 2022-05-11

**Authors:** Bahman Amani, Sara Zareei, Behnam Amani, Mahsa Zareei, Neda Zareei, Rouhollah Shabestan, Arash Akbarzadeh

**Affiliations:** ^1^ Department of Health Management and Economics, School of Public Health Tehran University of Medical Sciences Tehran Iran; ^2^ Department of Cell & Molecular Biology, Faculty of Biological Sciences Kharazmi University Tehran Iran; ^3^ Department of Health Services Management, School of Health Management and Information Sciences Iran University of Medical Sciences Tehran Iran; ^4^ Shiraz Transplant Research Center Shiraz University of Medical Sciences Shiraz Iran; ^5^ Department of Biostatistics and Epidemiology, School of Public Health Tehran University of Medical Sciences Tehran Iran

**Keywords:** artesunate, COVID‐19, imatinib, infliximab

## Abstract

**Background and Objective:**

Despite the pervasive vaccination program against coronavirus disease 2019 (COVID‐19), people who got fully vaccinated are still contaminated by severe acute respiratory syndrome coronavirus 2, making an effective and safe therapeutic intervention a crucial need for the patients' survival. The purpose of the present study is to seek available evidence for the efficacy and safety of three promising medications artesunate, imatinib, and infliximab against COVID‐19.

**Methods:**

A literature search was conducted in PubMed, Cochrane Library, medRxive, and Google Scholar, and the relevant articles published up to January 2022 were found. Furthermore, the clinical trial databases were screened for finding more citations. Data analysis was carried out applying The Cochrane Collaboration tool and Newcastle–Ottawa scale to assess the included studies. Meta‐analysis was performed using RevMan 5.4.1.

**Results:**

Five published studies were identified as eligible. Meta‐analysis showed that there was no significant difference between the infliximab and control groups in terms of mortality rate (risk ratio [RR]: 0.65; confidence interval [CI] 95%: 0.40–1.07; *p* = .09). However, a significant difference was observed between the two groups for the hospital discharge (RR: 1.37; CI 95%: 1.04–1.80; *p* = .03). No remarkable clinical benefit was observed for using imatinib in COVID‐19 patients. Artesunate showed significant improvement in patients with COVID‐19.

**Conclusion:**

In the present, limited evidence exists for the efficacy and safety of artesunate, imatinib, and infliximab in patients with COVID‐19. The findings of WHO's Solidarity international trial will provide further information regarding these therapeutic interventions.

## Graphical abstract

Our purpose was to review the available evidence of these three drugs in the treatment of coronavirus disease 2019 (COVID‐19). Our findings showed little evidence for the efficacy and safety of artesunate, imatinib, and infliximab in patients with COVID‐19.

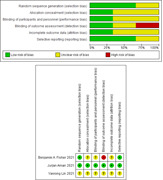

## 1 INTRODUCTION

Coronavirus disease 2019 (COVID‐19) is a global pandemic caused by the severe acute respiratory syndrome coronavirus 2 (SARS‐CoV‐2).^1^, ^2^ According to World Health Organization (WHO) report on September 8, 2021, there have been 221,648,869 confirmed cases of COVID‐19, including 4,582,338 deaths.^3^

To eradicate the infection, two main strategies have been followed by the medical communities around the world: The production of vaccines and finding a specific medicine. As of August 30, 2021, a total of 5,352,927,296 vaccine doses have been administered.^3^ However, there are still many nations where the pace of vaccination progress is slow. Moreover, it seems that the available vaccines may not guarantee patients' survival.^4^ What exacerbates the situation is the appearance of novel variants with higher transmission rates and evasion from the immunity created by vaccines or infection with the previous variants.^5^

In a condition where no specific medicine is approved by the Food and Drug Administration (FDA) against COVID‐19, using other drugs with probable efficiency might be the another strategy to contain the infection and decrease the death toll.^6^ Remdesivir was the first drug approved by the FDA for the treatment of hospitalized patients 12 years and older.^6^ However, the previous randomized clinical trial conducted by WHO Solidarity Trial Consortium found no remarkable clinical benefit of using remdesivir, hydroxychloroquine, lopinavir, and interferon in hospitalized patients with COVID‐19.^7^ Currently, WHO has announced a new phase in testing three drugs, including artesunate, imatinib, and infliximab in COVID‐19 treatment.^7^

Artesunate has been considered the first‐line treatment for cerebral and other severe types of malaria.^8^, ^9^ In addition, it has antibacterial,^10^ anti‐inflammatory,^11^ and antiallergic activities.^12^ The anticancer effect of artesunate has also been demonstrated for a variety of cancers including pancreatic cancer,^13^ ovarian cancer,^14^ leukemia,^15^ colorectal cancer,^16^ renal cell carcinoma,^17^ and lung cancer.^18^ More importantly, artesunate has antiviral effects against DNA and RNA viruses.^19^ Accordingly, artesunate can decrease the risk of death from Ebola,^20^ and has the highest antiviral activity against human cytomegalovirus compared with other derivatives of artemisinin.^21^ Regarding SARS‐CoV‐2 infection, Uzun et. al. proposed artesunate as an anti‐COVID‐19 agent because of the modulatory effect on inflammation and chloroquine‐like endocytosis.^22^ Moreover, in vitro experiments on A549‐hACE2 cells showed that artesunate is a potent agent against the virus after its entry,^23^ and in silico evaluations have shown that artesunate is capable of inhibiting the virus's main protease.^24^

Imatinib is a synthetic tyrosine kinase inhibitor used in the treatment of chronic myeloid leukemia,^25^, ^26^ chronic myelogenous leukemia,^27^ Philadelphia chromosome‐positive acute lymphoblastic leukemia,^28^ gastrointestinal stromal tumor,^29^, ^30^ hypereosinophilic syndrome,^31^ chronic eosinophilic leukemia,^32^ and systemic mastocytosis.^33^ Before the pandemic, its antiviral effects were demonstrated against SARS‐CoV, Middle East respiratory syndrome coronavirus 2, and infectious bronchitis virus (IBV) by in vitro studies.^34^, ^35^, ^36^ Imatinib drew attention after the emergence of SARS‐CoV‐2 again and some studies evaluated its potential benefits against COVID‐19 by clinical trials and in vivo/in vitro experiments.^37^, ^38^, ^39^ Although some studies claimed that imatinib may have potential effects against COVID‐19,^40^ others cast doubt on its efficacy.^41^, ^42^

Infliximab is a chimeric monoclonal antibody of human tumor necrosis factor‐α (TNF‐α) which is used in the treatment of intestinal Behçet's disease (BD),^43^, ^44^ Crohn's disease,^45^ ulcerative colitis,^46^, ^47^ rheumatoid arthritis,^48^ ankylosing spondylitis,^49^ nail psoriasis,^50^ moderate‐to‐severe plaque psoriasis,^51^ resistant psoriatic arthritis,^52^ refractory psoriasis including pustular psoriasis, and psoriatic erythroderma.^53^ Furthermore, it has been established that infliximab can reduce the mortality rate in COVID‐19 patients.^23^, ^39^

The aim of this rapid review was to provide the latest available evidence of three promising therapies, artesunate, imatinib, and infliximab against COVID‐19.

## 2 METHODS

We used the Preferred Reporting Items for Systematic reviews and Meta‐Analysis‐Rapid Review (PRISMA‐RR), a reporting guideline for rapid reviews of primary studies.^54^

### 2.1 Literature search strategy

A literature search was conducted in PubMed and Cochrane Library for the relevant records up to January 2022. In addition, Google Scholar, medRxiv, and clinical trial databases, including ClinicalTrials.gov, the European Union Clinical Trials Register, and the Chinese Clinical Trial Registry were searched for finding additional relevant documents. Finally, the reference list of the included studies was scanned to find more citations. The search was limited to articles with abstract and or full text in the English language. Search terms included 2019‐nCoV, SARS‐CoV‐2, COVID‐19, artesunate, imatinib, and infliximab.

### 2.2 Study selection

Two authors (Behnam Amani and Bahman Amani) independently screened the identified records based on inclusion and exclusion criteria. Disagreements between the authors were resolved by discussion among authors. The included inclusion were: (1) patients with confirmed positive COVID‐19 test; (2) artesunate, imatinib, and infliximab as monotherapy or in combination with other therapeutic agents; (3) any therapeutic intervention or placebo as a comparison (4); efficacy and safety outcomes of interest; and (5) clinical or observational studies. The exclusion criteria were the studies conducted on animal models, in vitro, in vivo, case reports, letters to editors, and editorials.

### 2.3 Data extraction and quality assessment

The Cochrane Collaboration tool was used to assess the risk of bias in randomized clinical trials.^55^ Quality assessment of observational studies was conducted using the Newcastle–Ottawa scale.^56^ Two authors (Rouhollah Shabestan and Mahsa Zareei) independently extracted data reported in the included studies using the same data extraction form. The extracted data included (1) study characteristics (author, year, setting, and design); (2) patient's characteristics (sample size, sex, and age); (3) intervention and comparison (sample size, dose, and treatment duration); and (4) efficacy and safety outcomes.

### 2.4 Evidence synthesis

We summarized the findings of the studies included in our review. For quantitative data, a meta‐analysis was performed using RevMan software, version 5.4.1. The risk ratio (RR) with a 95% confidence interval (CI) was used for dichotomous data. The random‐effects model was used for studies with *I* ^2^ > 50% or *p* < .1. Otherwise, the fixed‐effect model was used.

## 3 RESULTS

### 3.1 The characteristics of studies

**Figure 1:**
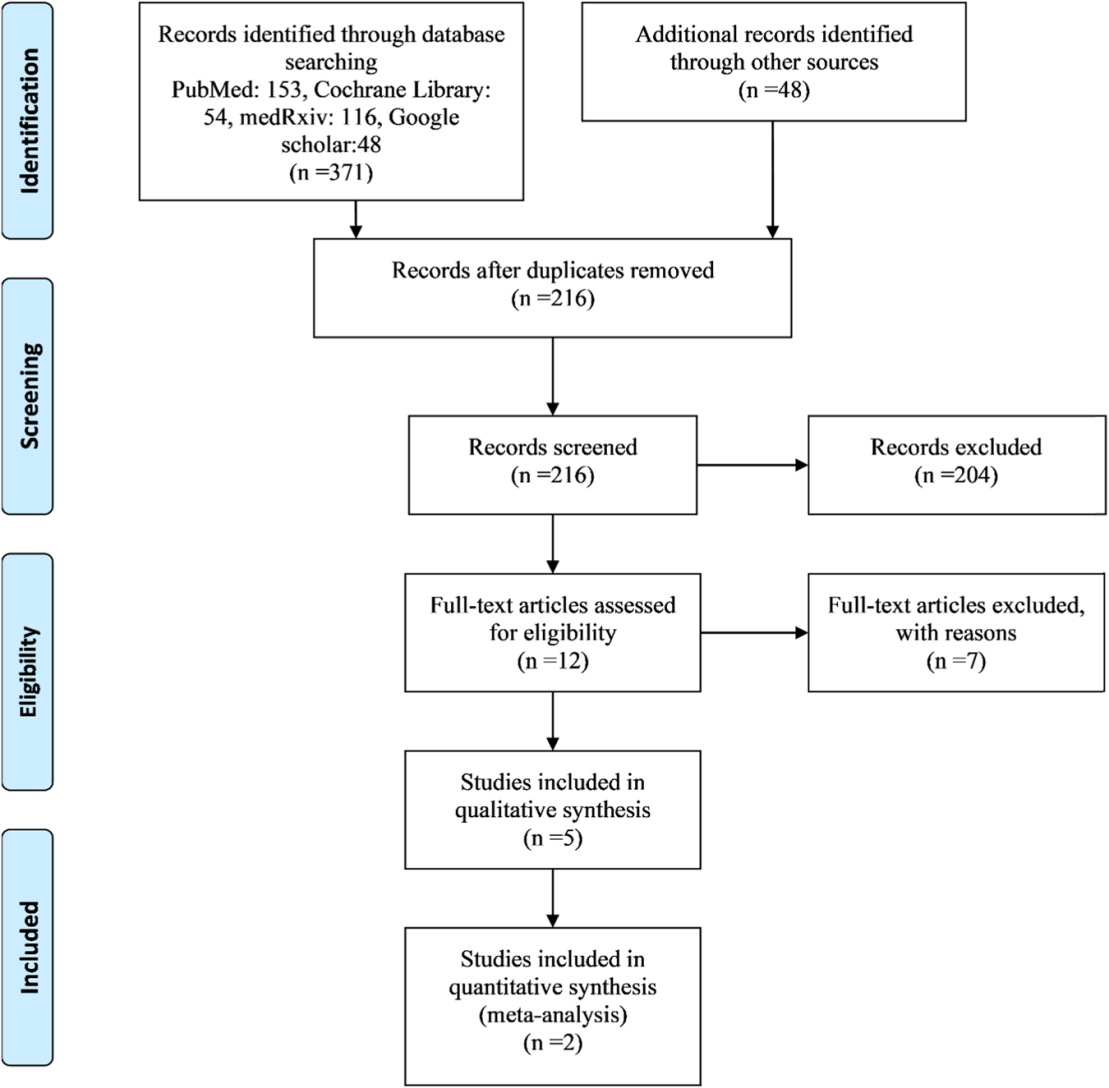
PRISMA flow diagram.

**Figure 2:**
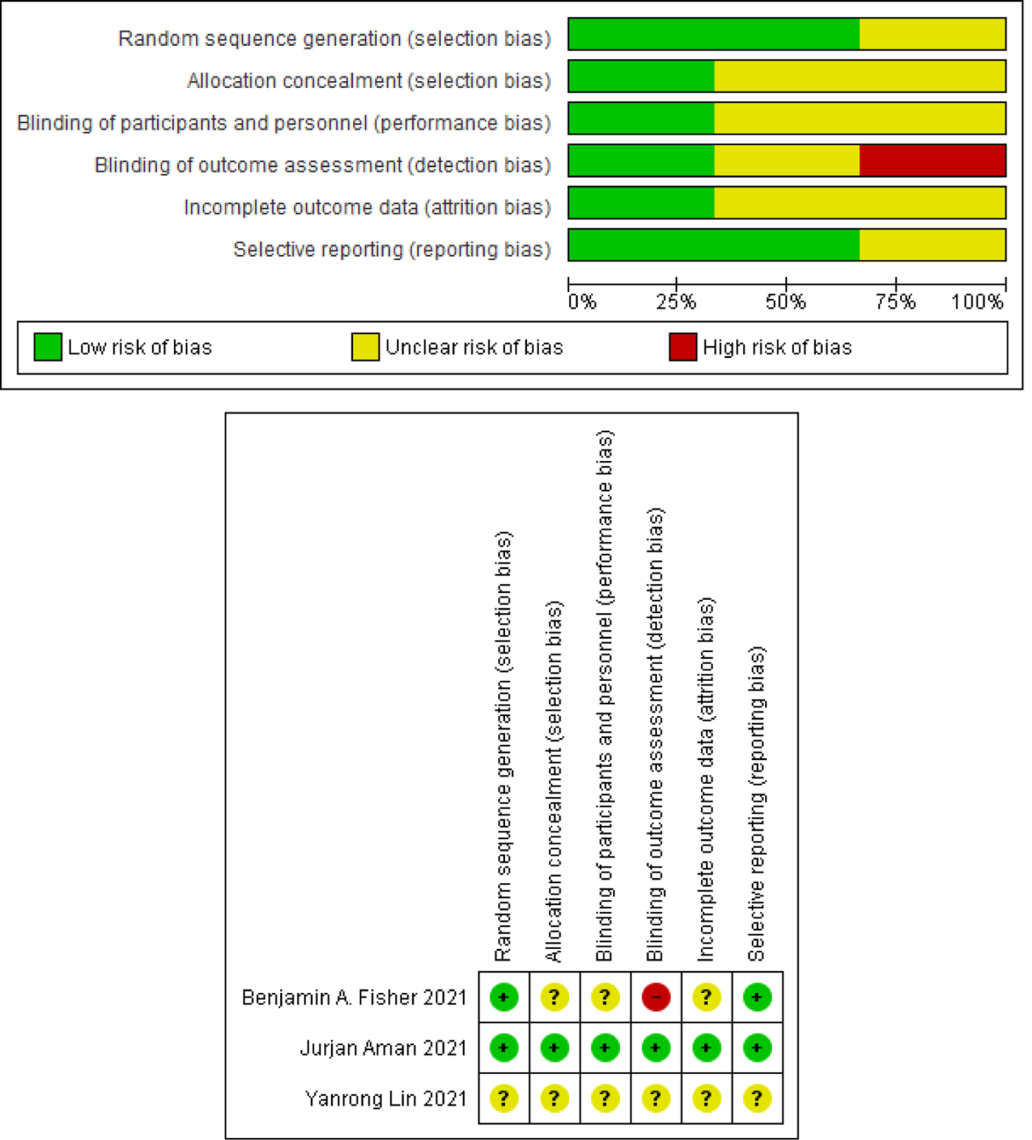
Risk of bias in the selected studies.

Figure 1 shows the literature search flow, removal of duplicates, and screening based on title, abstract, and full text. Out of eight studies in the full‐text step, five^42^, ^57^, ^58^, ^59^, ^60^ studies were considered for final analysis and their characteristics can be seen in Table 1. The risk of bias was determined using the Cochrane collaboration tool (Figure 2).

**Table 1:** The characteristics of included studies

Author, Year	Country	Design	*N*(M/F)	Intervention (*n*)	Comparison(s) (*n*)	Follow‐up
Farrokhpour, 2021^57^	Iran	Observational	104 (68/36)	Infliximab (27)	Control (43); IVIg (23); infliximab + IVIg (11)	NA
Fisher, 2021^58^	UK	RCT	146	Infliximab (35)	Usual care (54); namilumab (57)	28 days
Aman, 2021^42^	Netherlands	RCT	385 (264/121)	Imatinib (197)	Placebo (188)	28 days
Lin Y, 2021^59^	China	RCT	43	Artesunate (18)	Conventional (25)	10 days
Taleng, 2021^60^	USA	Observational	190 (50/140)	Infliximab (69)	Rituximab (121)	NA

Abbreviations: NA, not acquired; RCT, randomized clinical trial.

### 3.2 Artesunate

Only one study ^59^ examined the efficacy and safety of artesunate in patients with COVID‐19. Patients with confirmed COVID‐19 were divided into artesunate (*n* = 18) and conventional (*n* = 25) groups. Patients in the artesunate group received artesunate 60 mg, twice a day for 10 days. In the conventional group, patients received lopinavir/ritonavir 500 mg and interferon 500 U, twice a day for 10 days. The result showed a significant improvement in the duration of symptoms (3.33 ± 1.91 days vs. 4.84 ± 2.19 days; *p* < .05), polymerase chain reaction (PCR) negative conversion time (4.72 ± 2.16 days vs. 6.68 ± 3.76 days; *p* < .05), lung lesion absorption starting time (5.39 ± 2.36 days vs. 7.48 ± 3.78 days; *p* < .05), time of lung lesion absorption greater than 70% (14.11 ± 4.16 days vs. 17.04 ± 4.42 days; *p* < .05), and the length of hospital stay (16.56 ± 3.71 vs. 18.04 ± 3.97 days; *p* < .05) in patients received artesunate compared with conventional therapy, respectively. There was no significant difference between the two groups regarding adverse events (72.2% vs. 80.0%; *p* > .05).

### 3.3 Imatinib

In Aman et al.'s study,^42^ patients received either oral imatinib (*n* = 204) or placebo (*n* = 196). No significant difference was observed between the two groups in the stopping time of ventilation and supplemental oxygen support (adjusted hazard ratio [HR]: 1.07; 95% CI: 0.62–1.84; *p* = .82). In addition, no significant difference was observed between the two groups in terms of mortality rate (adjusted HR: 0.52; 95% CI: 0.26–0.95; *p* = .068) and the need for mechanical ventilation (adjusted HR: 1.02; 95% CI: 0.80–1.30; *p* = .87).

### 3.4 Infliximab

The meta‐analysis result showed that there was no significant difference in mortality rate between the infliximab and control groups (RR: 0.65; CI 95%: 0.40–1.07; *p* = .09). However, there was a significant difference between the groups in terms of hospital discharge (RR: 1.37; CI 95%: 1.04–1.80; *p* = .03). Farrokhpour et al.^57^ showed that there was no significant difference between infliximab and control regarding the length of hospital stay in hospitalized severe COVID‐19 patients in the intensive care unit (ICU). However, a significant difference was observed in patients receiving infliximab compared with the control group in terms of ICU admission duration.

In Phase 2 randomized adaptive trial conducted by Fisher et al.,^58^ the efficacy of infliximab in hospitalized patients with COVID‐19 was examined. Twenty out of 29 patients in the infliximab group were discharged compared with 22/34 in the usual care group. The WHO clinical progression scale in infliximab and usual care groups was 15 (6, 21 days) and 10 (6, 14 days) days, respectively. The frequency of adverse events was higher in infliximab patients (20/29) versus the usual care patients (17/34). Furthermore, infliximab was also compared with rituximab as an anticancer drug. It was established that patients prescribed by rituximab were more likely to manifest severe symptoms compared with those who received infliximab. However, the incidence of COVID‐19 in both treatments showed similar rates.^60^

## 4 DISCUSSION

The purpose of this study was to review the current evidence of the efficacy and safety of three promising therapies, artesunate, imatinib, and infliximab against COVID‐19. The findings of recently published systematic reviews and meta‐analyses found no sufficient and conclusive evidence for using hydroxychloroquine,^61^, ^62^ remdesivir,^63^ tocilizumab,^64^ lopinavir/ritonavir,^65^, ^66^ favipiravir,^67^ and arbidol^68^, ^69^ in COVID‐19 disease. Currently, artesunate, imatinib, and infliximab have been used on patients with COVID‐19.

There is little evidence regarding the treatment potential of these drugs. According to the findings of a study by Bae et al.,^70^ artesunate may have beneficial effects in patients with COVID‐19 and/or influenza. In Lin's study, artesunate administration was shown to be associated with improvement of symptoms, PCR negative conversion time, lung lesion absorption time, and the length of hospital stay in COVID‐19 patients.^59^ It is suggested that artesunate may function against SARS‐CoV‐2 by inhibiting viral S protein.^71^

The result of a randomized clinical trial^42^ showed imatinib was not superior to control in terms of discontinuation time of ventilation and supplemental oxygen, death, and the need for mechanical ventilation. Preclinical evaluation of imatinib by Touret et al.^72^ found no antiviral effect of imatinib against SARS‐CoV‐2. In addition, Zhao et al.^41^ indicated that imatinib had no inhibitory effect on SARS‐CoV‐2 infection. A recent in vitro study conducted by Lin et al. showed that imatinib can effectively prevent SARS‐CoV‐2 infection with low toxicity.^73^ Morales‐Ortega et al.^40^ in a study monitored the COVID‐19 patients treated with imatinib. Seventeen of 20 patients recovered and three patients died. Adverse events were reported mild among patients.

The findings of one study found no benefit for using infliximab in COVID‐19 patients in terms of the length of hospital stay.^74^ According to the findings of meta‐analysis, patients taking infliximab showed earlier hospital discharge compared with the control group. Nonetheless, infliximab showed no benefit in terms of mortality rate. However, these findings should be interpreted with caution due to patients in the control groups received different treatment protocols.

## 5 LIMITATIONS

The limitations of the present studies include the finite number of studies, small sample size, and low methodological quality of studies. In addition, due to the small sample size in this study, the efficacy of artesunate needs to be further explored and verified. Further studies are required to validate these findings.

## 6 CONCLUSION

**Figure 3:**
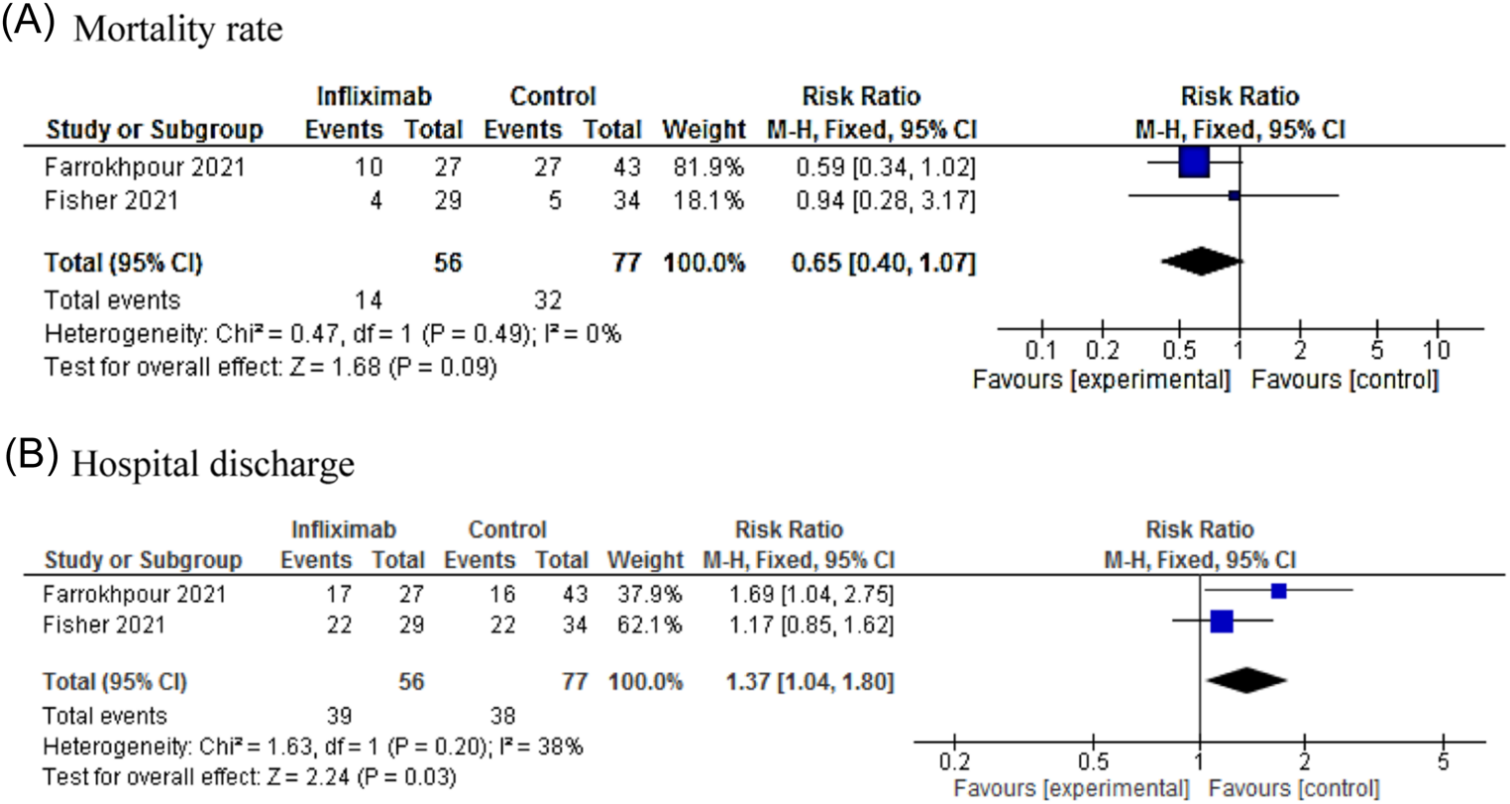
Forest plot of infliximab versus control for mortality rate (A) and hospital discharge (B). CI, confidence interval

Artesunate showed better efficacy in the improvement of symptoms, PCR negative conversion time, lung lesion absorption time, and the length of hospital stay compared with the control group. Even though we find no evidence for imatinib's antiviral impact, it may still hold promise for the treatment of COVID‐19 since limited evidence is available for downright dismissal of imatinib. The result of the meta‐analysis showed no clinical benefit for infliximab in terms of mortality rate. Further evidence is needed to assert the efficacy and safety of these therapeutic agents in COVID‐19 improvement. The result of WHO's Solidarity international trial will provide further information regarding the therapeutic effects of artesunate, imatinib, and infliximab in hospitalized patients with COVID‐19 (Figure 3).

## AUTHOR CONTRIBUTIONS

*Conceptualization, project administration, and supervision*: Bahman Amani and Behnam Amani. *Formal analysis and Software*: Bahman Amani, Behnam Amani, and Arash Akbarzadeh. *Investigation*: Bahman Amani, Behnam Amani, Sara Zareei, Mahsa Zareei, and Neda Zareei. *Methodology*: Arash Akbarzadeh, Behnam Amani, Bahman Amani, Rouhollah Shabestan, and Mahsa Zareei. *Writing—original draft*: Behnam Amani. *Writing—review & editing*: Behnam Amani, Sara Zareei, and Bahman Amani. *Data collection*: Mahsa Zareei and Neda Zareei.

## CONFLICTS OF INTEREST

The authors declare no conflicts of interest.

## Data Availability

The data that support the findings of this study are openly available in these studies.^42^, ^57^, ^58^, ^59^, ^60^
